# Amlexanox Enforces Osteogenic Differentiation and Bone Homeostasis Through Inhibiting Ubiquitin-Dependent Degradation of β-Catenin

**DOI:** 10.7150/ijbs.101507

**Published:** 2024-09-30

**Authors:** Qian He, Zhouboran Liu, Xuan Xia, Jun Zeng, Yuling Liu, Jingqiong Xun, Meilu Liu, Yueming Mei, Ruchun Dai

**Affiliations:** 1 National Clinical Research Center for Metabolic Diseases, Hunan Provincial Key Laboratory of Metabolic Bone Diseases, and Department of Metabolism and Endocrinology, The Second Xiangya Hospital of Central South University, Changsha 410011, P.R. China.; 2 Department of Physiology and Pathophysiology, College of Basic Medical Sciences, China Three Gorges University, Yichang 443002, P.R. China.; 3 Department of Endocrinology, The First College of Clinical Medical Science, China Three Gorges University & Yichang Central People's Hospital. Yichang 443002, P.R. China.; 4 Department of Endocrinology, Guizhou Provincial People's Hospital. Guiyang 550002, P.R. China.; 5 Hubei Key Laboratory of Tumor Microenvironment and Immunotherapy, China Three Gorges University, Yichang 443002, P.R. China.

**Keywords:** Amlexanox, BMSCs, β-catenin, IKKε, Ubiquitination, Osteoporosis.

## Abstract

There was arising osteoporosis from an imbalance in bone remodeling, with excessive differentiation of bone marrow mesenchymal stem cells (BMSCs) into adipocytes instead of osteoblasts. In this study, we found IKKε was upregulated in osteoporotic bone and *Ikbke* knockdown promoted osteoblast differentiation. We explored amlexanox (AM), a novel IKKε inhibitor, for its effects on osteogenic differentiation and bone homeostasis. AM treatment in mice decreased bone loss, reduced marrow fat, and improved bone microarchitecture, leading to enhanced bone strength. In vitro, AM promoted osteogenesis and suppressed adipogenesis of BMSCs in a dose-dependent manner. Moreover, AM controlled RANKL/OPG expression of BMSC which regulated osteoclast differentiation. Mechanistic explorations revealed AM reinforced Wnt/β-catenin pathway by suppressing ubiquitin-proteasome-dependent degradation of β-catenin. Importantly, AM stimulated osteogenesis in human BMSCs. By promoting osteogenesis at the expense of adipogenesis and hindering osteoclastogenesis, AM offers a promising therapeutic strategy for osteoporosis due to its established safety profile.

## Introduction

Osteoporosis is characterized by a decline in bone formation and a concurrent increase in bone marrow fat content^1^-^3^. Bone marrow mesenchymal stem cells (BMSCs) possess the unique ability to differentiate into various cell types, including osteoblasts, adipocytes, and chondrocytes under specific signals^4^. However, in osteoporosis, BMSCs exhibit a shift in their differentiation preference, favoring adipocyte formation over osteoblast development^5^. This imbalance is considered a critical contributor to the pathogenesis of osteoporosis. Elucidating the precise molecular mechanisms governing the BMSC switch from osteogenesis to adipogenesis underpins a deeper understanding of osteoporosis development and facilitates the development of novel therapies to promote bone formation and combat this disease.

Amlexanox (AA-673, AM), a synthetic pyridine-3-carboxylic acid derivative developed in the 1980s, was initially shown to inhibit immune response and act as a leukotriene antagonist with anti-asthma effects in animal models^6^, ^7^. From 1990s, AM emerged as a widely-used oral paste, Food and Drug Administration (FDA)-approved for treating recurrent aphthous ulcer (RAU) in clinics across nations like America and China^8^, ^9^. Recent research suggested AM inhibited IκB kinase epsilon (IKKε) and TANK-binding kinase 1 (TBK1), implicated in various diseases including non-alcoholic fatty liver disease (NAFLD), type 2 diabetes, obesity, rheumatoid arthritis, and tumor^10^-^13^. Both basic research and clinical trials demonstrated that AM significantly decreased blood glucose and body weight, and improved insulin sensitivity, dyslipidemia, and hepatic steatosis^14^-^16^. These findings suggest development potential for AM and its analogs in treating metabolic syndrome.

IKKε (Gene name: *Ikbke*), a noncanonical IKK in the NF-κB upstream signaling pathway, is induced by lipopolysaccharide (LPS)^17^. However, it also plays a crucial role in interferon (IFN) signaling, essential for fighting viral infections^18^. While the role of IKKε in bone diseases was emerging, research focused on synoviocyte-mediated inflammation in rheumatoid arthritis (RA) and osteoclast-mediated bone resorption. Clinical studies linked IKKε activity to RA severity, with IKKε promoting nociception and inflammation through the NF-κB pathway^11^, ^19^. Interestingly, IKKε inhibition in breast cancer cells reduced their growth, migration, and ability to enhance osteoclastogenesis by impacting both interferon regulatory factor (IRF) and NF-κB pathways^20^. However, the role of IKKε in osteogenesis remains unclear, and further investigation is needed to determine whether AM can be used to treat osteoporosis by improving the osteoblastic function of BMSCs.

Our study revealed that IKKε expression was increased in osteoporotic bone tissue and *Ikbke* negatively regulated osteogenesis. AM treatment reduced bone loss, decreased bone marrow fat, and improved bone microarchitecture, ultimately leading to enhanced bone strength in ovariectomized (OVX) mice. Mechanistically, AM promoted osteogenesis and suppressed adipogenic differentiation of mouse bone marrow stem cells (mBMSCs) in a dose-dependent manner. Additionally, it regulated osteoclast differentiation by affecting RANKL and OPG expression in mBMSCs. Further investigation revealed that AM strengthened the Wnt/β-catenin signaling pathway by inhibiting ubiquitin-proteasome-dependent degradation of β-catenin. Importantly, AM also promoted osteogenesis in human bone marrow stem cells (hBMSCs).

## Materials and methods

### Bioinformatics analysis

*IKBKE* expression data was downloaded from the Human Protein Atlas (HPA) database (proteinatlas.org) and analyzed across 40 normal human tissues. Microarray data (GSE35959) related to osteoporosis was obtained from the Gene Expression Omnibus (GEO) database (https://www.ncbi.nlm.nih.gov/gds). This dataset included mRNA expression profiles from four human MSCs derived from non-osteoporotic donors and five MSCs from osteoporosis patients. Differentially expressed genes (DEGs) were identified using the GEO2R online tool with the following filtering criteria: |log2-fold change| ≥ 1 and adjusted P-value (adj. P) < 0.05.

### Animals and amlexanox treatment

This animal study was approved by the Animal Care Committee of Second Xiangya Hospital of Central South University (No. 20220163) and adhered to National Institutes of Health's Guide for the Care and Use of Laboratory Animals.

Twenty-two eight-week-old female C57BL/6J mice were obtained from the Laboratory Animal Center of the Second Xiangya Hospital of Central South University. Mice were housed under controlled conditions (12-hour light/dark cycle, 23±2 °C, 55±10% humidity) in a specific pathogen-free environment. Ikbke^fl/fl^ mice and Prx1-Cre transgenic mice were produced from the Cyagen Bioscience (Jiangsu, China). Ikbke^fl/fl^ mice were crossed with Prx1-Cre mice to generate mice with conditional knockout of *Ikbke* specifically in BMSC (Prx1-Cre; Ikbke^fl/fl^). The Ikbke^fl/fl^ mice were used as controls.

Mice were anesthetized and randomly assigned to four groups: 1) Sham group: Underwent a sham operation and received vehicle treatment (gavage, once daily); 2) OVX group: Underwent bilateral ovariectomy surgery and received vehicle treatment (gavage, once daily); 3) AM Low Dosage (AM-LD) group: Underwent ovariectomy surgery and received daily intragastric administration of AM (50 mg/kg body weight) dissolved in Tris-HCl buffer (250 mM Tris-HCl buffer, pH 7.2, adjusted with 150 mM sodium hydroxide); 4) AM High Dosage (AM-HD) group: Underwent ovariectomy surgery and received daily intragastric administration of AM (100 mg/kg body weight) dissolved in Tris-HCl buffer (as above). AM (Abcam, USA) was administered to the mice for eight weeks, followed by euthanasia under isoflurane anesthesia. Uteri were dissected and weighed to confirm ovariectomy efficacy. Femora and tibias were harvested for further analysis.

### Micro-CT (μCT) analysis

Right femora were fixed in 4% paraformaldehyde and analyzed using high-resolution micro-CT (Skyscan 1176, Bruker, Belgium) for trabecular and cortical bone analysis. Scanning parameters (100 μA current, 80 kV voltage) yielded a spatial resolution of 9 μm. Image reconstruction (Data Viewer v1.5) and data analysis (CTAn v1.13) were performed using dedicated software.

For trabecular bone, a region of interest (ROI) was defined at 5-10% of the femoral length from the distal growth plate. The following parameters were quantified: trabecular volumetric bone mineral density (vBMD, g/cm^3^), trabecular bone volume fraction (Tb.BV/TV, %), trabecular bone surface density (Tb.BS/TV, per mm), trabecular number (Tb.N, per mm), trabecular thickness (Tb.Th, per mm), trabecular spacing (Tb.Sp, per mm), structure model index (SMI) and connectivity density (Conn.Dn, per mm^3^).

For cortical bone, the ROI was set at 20-30% of the femoral length from the growth plate. Cortical area fraction (Ct.Ar/Tt.Ar, %) and cortical bone thickness (Ct.Th, per mm) were quantified.

### Biomechanical test

The mechanical properties of the femora were evaluated using a three-point bending test performed on an electronic universal testing machine (WDW3100, Changchun Testing Machine Institute, Changchun, China). The femora were placed on two horizontal supports with a 7 mm span between them. A vertical loading was applied at the midpoint at a constant rate of 1 mm/min until fracture occurred. The load-displacement curve was recorded, and the following biomechanical properties were calculated from the curve: maximum bending load (N), maximum bending strength (MPa), stiffness constant K (N/mm) and elastic modulus (MPa).

### Calcein double-labeling assay

Mice were treated with calcein (10 mg/kg; Sigma-Aldrich, USA) in PBS by intraperitoneal injection at 10 days and 3 days before sacrifice. Tibias were fixed in 4% paraformaldehyde for 48h. Undecalcified bone sections (10 μm thick) were prepared and visualized using a fluorescence microscope (Carl Zeiss, Germany). The trabecular bone mineral apposition rate (MAR) was quantified by analyzing three random fields per tibia.

### Histological and immunohistochemical staining

Dissected femurs were fixed (4% paraformaldehyde, 2 days) and decalcified (10% EDTA, 3 weeks) before processing for paraffin embedding and sectioning (6 μm). Hematoxylin and eosin (H&E) staining was performed according to standard protocol. For immunohistochemistry (IHC), deparaffinized sections underwent antigen retrieval and blocking with 5% BSA for 1 hour. Sections were then incubated overnight at 4°C with primary antibodies against osteocalcin (OCN, bs-4917R, Bioss, China) and β-catenin (51067-2-AP, Proteintech, China). After incubation with HRP-conjugated secondary antibody (Boster, China) for 1 hour, immunoreactivity was visualized using a DAB chromogenic substrate kit (Boster, China). Finally, sections were counterstained with hematoxylin and visualized under a light microscope (Carl Zeiss, Germany).

### Isolation, culture and characterization of BMSCs

Mouse BMSCs (mBMSCs): Primary mBMSCs were isolated from femurs and tibias of 6- to 8-week-old mice and cultured in Minimum Essential Medium α (α-MEM) supplemented with 10% fetal bovine serum (FBS) and 1% penicillin and streptomycin (P/S, Gibco, USA).

Human BMSCs (hBMSCs): hBMSCs were isolated as previously described^21^ following approval from the Ethics Committee of the Second Xiangya Hospital of Central South University (No. LYF 2023049). Briefly, bone marrow aspirates from 6 osteoporosis patients undergoing spinal fusion surgery (details in Supplementary Table 1) were centrifuged (1200 rpm, 5 min). Cells were cultured in complete low-glucose Dulbecco's modified Eagle's medium (DMEM) (Gibco, USA) with 10% FBS and 1% P/S. Non-adherent cells were removed after 96 hours. hBMSCs at passage 3 or 4 were used for experiments.

Characterization of BMSCs: Both mouse and human BMSCs were characterized by flow cytometry analysis of specific surface markers. For mBMSCs, these markers included CD44-PE (Biolegend, USA), CD29-PE (ThermoFisher, USA), Sca-1-PE/Cy7 (Elabscience, China), CD31-PerCP/Cy5.5 (Elabscience, China), and CD19-BV711 (ThermoFisher, USA). Human BMSCs were analyzed for CD105-APC (BD Pharmingen, USA), CD44-PE (Biolegend, USA), CD14-FITC (BD Pharmingen, USA), and CD34-PE (BD Pharmingen, USA) expression.

### Cell culture and treatments

C3H10T1/2 and Raw 264.7 were cultured in DMEM (Gibco, USA). MC3T3-E1 was cultured in α-MEM. All culture media were supplemented with 10% FBS and 1% P/S (Gibco, USA).

For osteogenic differentiation, BMSCs were cultured in the osteogenic induction medium (α-MEM or DMEM supplemented with 10% FBS, 1% P/S, 50 μM L-ascorbic acid, 10 mM β-glycerophosphate and 0.1 μM dexamethasone). The osteogenic induction medium was changed every 3 days. After 7 or 14 days of osteogenic induction, cells were assessed for osteogenic differentiation using alkaline phosphatase (ALP) staining or Alizarin Red S (ARS) staining, respectively. AM (ab142825, Abcam, USA) was prepared as a 100 mM stock solution in DMSO and stored at -20°C. To investigate its effects on osteogenesis, various concentrations of AM were added to the osteogenic media to study their impact on osteogenesis.

For adipogenic differentiation, BMSCs were cultured in adipogenic induction medium (α-MEM or DMEM supplemented with 10% FBS, 1% P/S, 10 μg/ml insulin, 0.5 mM 3-isobutyl-1-methylxanthine and 1 μM dexamethasone). The medium was changed every 2 days until the cells were ready for Oil Red O staining.

### Osteoclast differentiation assay

Raw 264.7 cells (2.5 x 10^5^ cells/well) were seeded in 6-well plates with α-MEM. For osteoclast induction, cells were treated with conditioned medium containing 100 ng/mL receptor activator for nuclear factor κB ligand (RANKL, PeproTech, USA). Osteoclasts were identified by tartrate-resistant acid phosphatase (TRAP) staining.

Preparation of the conditioned medium (CM): BMSCs were treated with or without 1.5 μM AM (AM) during osteogenic induction. After 7 days, the medium was replaced with fresh complete medium for an additional 2 days. CM from vehicle-treated (BMSC^Veh^-CM) and AM-treated BMSCs (BMSC^AM^-CM) were collected, centrifuged (3000g, 10 min), and used to induce osteoclast differentiation in Raw 264.7 cells.

### Lentivirus infection

2x10⁵ BMSCs were seeded in 6-well plates and transduced with Control-shRNA or Ikbke-shRNA lentivirus (Genechem, China) for 16 hours. After 72 hours, green fluorescence was observed under a fluorescence microscope (Carl Zeiss, Germany) to assess transfection efficiency. Subsequently, the culture medium was replaced with osteogenic induction medium for 7 or 14 days.

### siRNA-mediated β-Catenin knockdown and cell transfection

Ctnnb1 siRNA (Ctnnb1 siRNA-1: GCACCATGCAGAATACAAA; Ctnnb1 siRNA-2: GAATGAGACTGCAGATCTT; Ctnnb1 siRNA-3: CAAGCCTTAGTAAACATAA) and a negative control siRNA (siCtrl) (RiboBio, China) were used to knock down β-catenin expression. Transfection of siRNA oligonucleotides was performed using HiPerFect (Qiagen, Germany) according to the manufacturer's instructions. For analysis of the osteogenic differentiation capacity, transfection medium was removed after 8 hours and replaced by osteogenic induction medium.

### Nuclear protein extraction and western blotting

Cells were lysed in RIPA buffer (Beyotime, China) and protein concentration was determined using a BCA assay kit (Boster, China). Nuclear proteins were isolated from cell lysates using a commercially available Nuclear and Cytoplasmic Protein Extraction Kit (Beyotime, China) following the manufacturer's protocol. Equal amounts of protein lysates were boiled with loading buffer, separated by SDS-PAGE (10% sodium dodecyl sulfate-polyacrylamide gel), and transferred to polyvinylidene difluoride (PVDF) membranes. Membranes were blocked with 5% skim milk in TBST for 2 hours at room temperature and then incubated overnight at 4°C with primary antibodies against IKKε (sc-376114, Santa Cruz, USA), RUNX2 (bs-1134R, Bioss, China), ALP (sc-271431, Santa Cruz, USA), COL1α (67288-1-Ig, Proteintech, China), PPARγ (sc-7273, Santa Cruz, USA), FABP4 (sc-271529, Santa Cruz, USA), β-catenin (51067-2-AP, Proteintech, China) and GAPDH (60004-1-Ig, Proteintech, China). HRP-conjugated secondary antibodies specific to the primary antibody species were used. Protein bands were visualized using ECL reagents (Millipore, USA) and imaged with an Amersham Imager 600 system (GE, USA).

### RNA isolation and quantitative real-time reverse transcriptase polymerase chain reaction (qRT-PCR)

Total RNA was extracted from cells with TRIzol reagent (Invitrogen, Carlsbad, CA). Bone tissues were ground into powder in liquid nitrogen and then TRIzol was added. Complementary DNA (cDNA) was synthesized from the isolated RNA using the PrimeScript RT reagent kit (RR047A, TaKaRa, Japan) according to the manufacturer's protocol. qRT-PCR was performed using TB Green Premix Ex Taq (RR820A, TaKaRa, Japan) in a 20 μL reaction volume. The thermal cycling conditions were: initial denaturation at 95°C for 30 seconds, followed by 40 cycles of denaturation at 95°C for 5 seconds and annealing/extension at 60°C for 30 seconds. The relative expression of mRNA was calculated by the 2^-△△Ct^ method and normalized to GAPDH expression. The designed primers sequences for mouse and human genes were provided in Table 1 and 2, respectively.

### Immunocytofluorescence assay

The cells were fixed with 4% paraformaldehyde for 30 min, permeabilized with 0.5% Triton X-100 for 10 min, and blocked with 1% bovine serum albumin for 15 min. Subsequently, cells were incubated overnight at 4°C with primary antibodies against active-β-catenin (Millipore, USA). This was followed by incubation with a fluorescence-conjugated secondary antibody (Proteintech, China) for 1 hour and with 4',6-diamidino-2-phenylindole (DAPI, Solarbio, China) for 5 minutes to stain the nuclei. The samples were visualized by fluorescence microscope (Carl Zeiss, Germany).

### TOP/FOP flash reporter assay

293T and C3H10T1/2 cells were seeded in 12-well plates and cultured for 12 hours. The cells were then transfected with luciferase reporter plasmids: 0.5 μg/well of either TOP flash (D2505, Beyotime, China) or FOP flash (D2507, Beyotime, China) and 10 ng of Renilla luciferase plasmid (pRL-TK, Promega, USA). GP transfect mate (G04008, GenePharma, China) was used as a transfection reagent. To assay the β-catenin activity after AM treatment, cells were stimulated with DMSO or AM (1.5 μM) 24 hours after transfection. Subsequently, a dual-luciferase reporter assay kit (#E1960, Promega, USA) was used to measure firefly and Renilla luciferase activity. The ratio of firefly luciferase (driven by β-catenin-mediated TCF binding) to Renilla luciferase (normalization control) reflects β-catenin activity.

### Cycloheximide chase assay

Cycloheximide (CHX) (Selleck, UK) and MG-132 (Beyotime, China) were dissolved in DMSO and stored at -20°C. 3-Methyladenine (3-MA, Selleck, UK) was suspended in PBS and prepared for immediate use. Cells were pre-treated with AM for 24h and then incubated in combination with CHX (50 μg/mL) at different time gradients. To elucidate the proteasome-dependent manner, cells pre-treated with AM were intervened with CHX alone or in combination with MG132 (20 mM). Then cells pre-treated with AM were co-incubated with or without 3-MA in the presence of CHX (5 mM).

### Immunoprecipitation and ubiquitylation assay

For immunoprecipitation experiments, cells were incubated with 20 mM MG-132 (Beyotime, China) for 8 hours to block the proteasome-mediated degradation. Whole-cell lysates were prepared using Cell lysis buffer (Beyotime, China) supplemented with protease inhibitors (Cwbio, China) and PMSF (Beyotime, China). Lysates were incubated with anti-β-catenin antibody (51067-2-AP, Proteintech, China) overnight at 4°C, followed by Protein A+G Agarose beads (Beyotime, China) for 3 hours at 4°C. After washing with ice-cold PBS (5 times), immunoprecipitated proteins were eluted with 1×loading buffer (Beyotime, China) by boiling at 100°C for 5 minutes. Immunoblots were probed with the same anti-β-catenin antibody or anti-ubiquitin monoclonal antibodies (sc-8017, Santa Cruz, USA) to assess β-catenin ubiquitination.

PR-619 Treatment: Cells were co-incubated with 15 μM PR-619 (HY-13814, MedChemExpress, China) in the presence or absence of AM for 48 hours. Pull-down proteins were resolved by SDS-PAGE and immunoblotted with anti-β-catenin or anti-ubiquitin antibodies.

### Statistical analysis

Data were presented as mean ±standard deviation (SD). Statistical analyses were performed using GraphPad Prism version 9.0 (San Diego, USA). Unpaired Student's t-test was used for comparisons between two groups. One-way or two-way ANOVA followed by Bonferroni's post hoc test was used for multiple comparisons. Statistical significance was set at P < 0.05.

## Results

### IKKε expression was upregulated in osteoporotic bone tissue and downregulated during osteogenic differentiation

**Figure 1:**
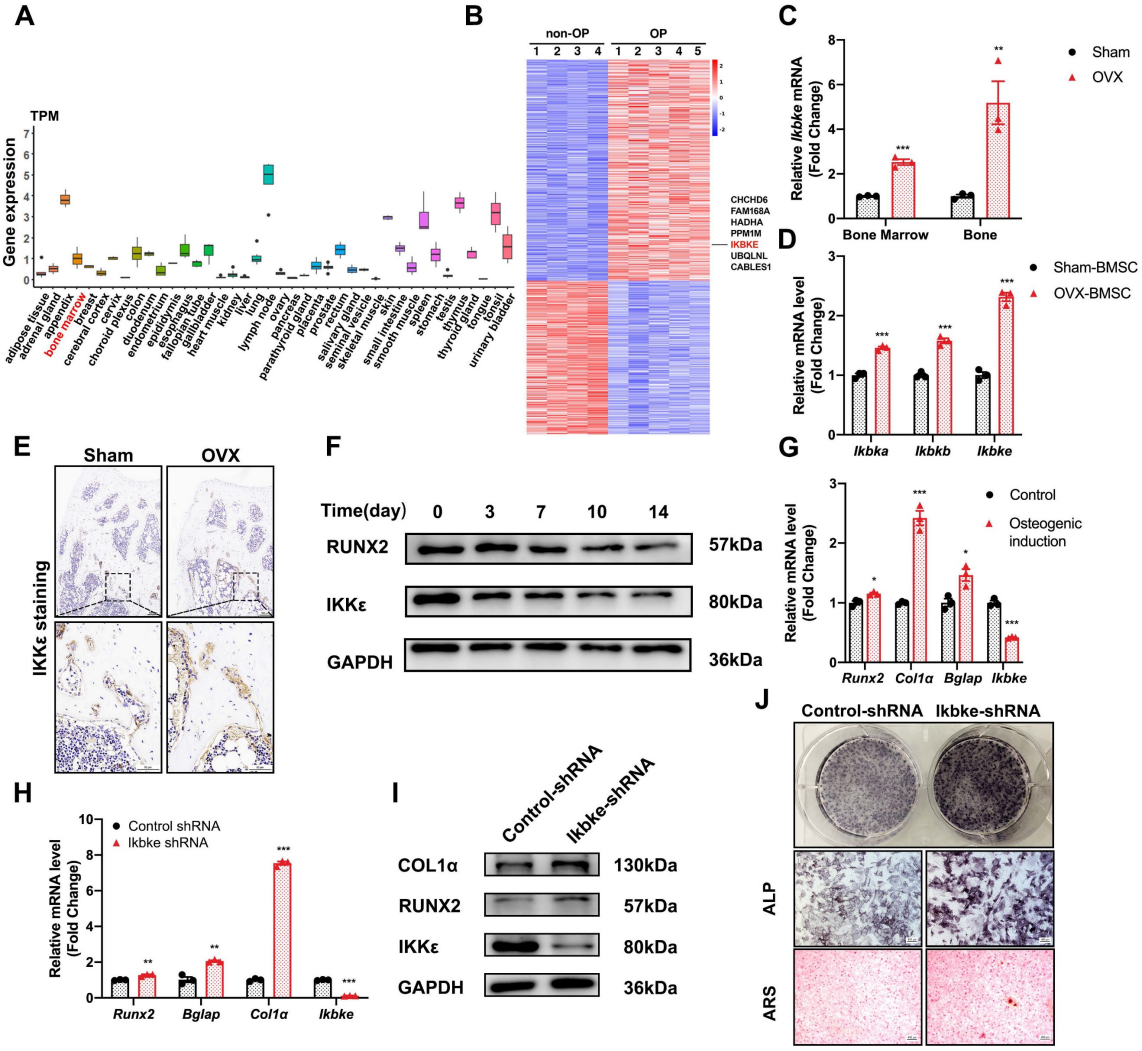
** IKKε expression was upregulated in osteoporotic bone tissue and downregulated osteogenic differentiation.** (A) *IKBKE* expression pattern in 40 human normal tissues from Human Protein Atlas Dataset. (B) Heatmap of DEGs in microarray data (GSE35959). (C) IKKε expression examined by qRT-PCR in bone and bone marrow from sham or OVX mice (n=3). (D) qRT-PCR analysis of IKK members. (E) Representative images of immunohistochemical staining of IKKε. Scale bar: 100 μm (upper panels) and 50 μm (lower panels). (F) Western blot analysis of IKKε in mBMSCs during osteogenesis differentiation (n=3). (G) Osteogenic genes including *Runx2, Col1α, Bglap* and *Ikbke* were measured by qRT-PCR in mBMSC. (H-J) mBMSCs underexpressing* Ikbke* were induced to allow osteogenic differentiation. The effects of *Ikbke* silencing on osteoblast differentiation were examined. The mRNA (H) and protein levels (I) of osteogenic factors expression were measured. ALP and ARS staining of mBMSC (J). Scale bar= 400 μm. Data are presented as mean ± SD. *P < 0.05; **P< 0.01; ***P < 0.001, Student's t test. OVX, ovariectomy; ALP, Alkaline phosphatase; ARS, alizarin red S.

We examined *IKBKE* expression patterns across various human tissues and found moderate levels in bone marrow (Fig. 1A). Interestingly, analysis of hBMSCs revealed elevated *IKBKE* expression in cells derived from osteoporosis patients (Fig. 1B). This finding prompted further investigation in OVX mouse model, a well-established model for osteoporosis (Fig. S1). Immunohistochemical analysis confirmed elevated IKKε levels in bone tissue from OVX mice (Fig. 1E). Similar results were observed in bone marrow and bone from OVX mice, showing a significant increase in *Ikbke* expression compared to sham mice (Fig. 1C). Importantly, *Ikbke* expression was significantly higher in OVX-derived mBMSCs compared to other IKK family members (Fig. 1D). Additionally, proliferation of OVX-derived mBMSCs was impaired (Fig. S1C).

Following osteogenic induction, mBMSCs from OVX mice displayed significantly reduced ALP staining (Fig. S2E). Furthermore, OVX-derived mBMSCs exhibited decreased expression of key osteogenic genes (RUNX2 and COL1α) compared to sham, coinciding with increased IKKε expression (Fig. S2F). We investigated IKKε expression in mBMSCs throughout the 14-day osteogenic differentiation process. Compared to undifferentiated cells, differentiated mBMSCs showed a progressive decrease in IKKε protein and mRNA levels (Fig. 1F, 1G, S2D and S2H). A similar decline in *Ιkbke* was observed in the osteoblast cell line MC3T3-E1 after osteogenic induction for 14 days (Fig. S2G). Overall, these findings demonstrated that IKKε expression was elevated in BMSCs from individuals with osteoporosis, suggesting a potential negative role for IKKε in bone formation.

### Silencing of Ikbke stimulated osteoblast differentiation

To confirm the role of *Ikbke* in osteogenesis, lentivirus-mediated shRNA was used to knock down *Ikbke* in mBMSCs. ShRNA-2 effectively silenced* Ikbke*, as confirmed by significant reductions in both mRNA and protein levels compared to the control group (Fig. S2A and S2B). In loss-of-function study, the knockdown of *Ikbke* in mBMSCs stimulated osteogenic differentiation, which was evidenced by the enhancement of ALP and ARS staining (Fig. 1J). Furthermore, qRT-PCR revealed elevated expression of osteogenic marker genes (Runx2, Alp, Col1α, and Bglap) (Fig. 1H), and Western blot confirmed increased protein levels of RUNX2 and COL1α (Fig. 1I and S2C). These findings collectively demonstrated that Ikbke knockdown promoted osteogenic differentiation in BMSCs.

### Amlexanox enhanced bone formation, reduced marrow adiposity, and improved bone microarchitecture in OVX mice

**Figure 2:**
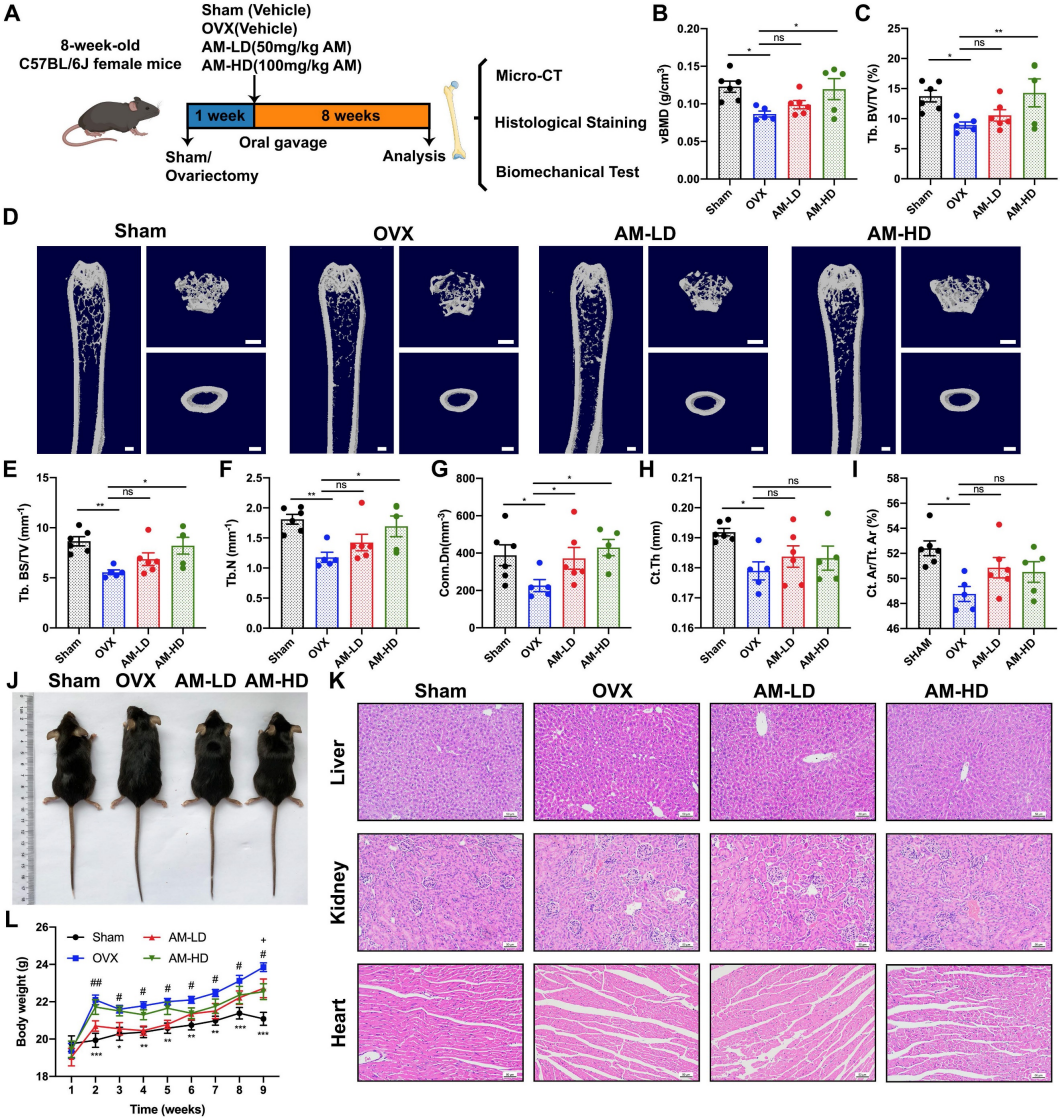
** Amlexanox reduced ovariectomy-induced bone loss and improved bone microarchitecture.** (A) Schematic diagram of the *in vivo* experiments. (D) Representative μCT images showed the femur bone loss was prevented by AM administration. Scale bar= 500 μm. (B, C, and E-I) Quantitative analyses of parameters regarding bone microstructure, including vBMD, Tb.BV/TV, Tb.BS/TV, Tb.N, Conn.Dn, Ct. Th and Ct. Ar/Tt. Ar. *P < 0.05; **P< 0.01; ***P < 0.001, one-way ANOVA. (J) Representative images of mouse morphology in each group. (K) H&E staining of vital organs. Scale bar= 50μm. (L) The body weight. *P < 0.05, **P< 0.01, ***P < 0.001, compared with OVX; ^#^*P* < 0.05, ^##^*P*< 0.01, compared with AM-LD;^ +^*P* < 0.05, compared with AM-HD, two-way ANOVA. Data are presented as mean ± SD. n=5 or 6 per group. vBMD, volumetric bone mineral density; Tb. BV/TV, trabecular bone volume fraction; Tb.BS/TV, trabecular bone surface density; Tb.N, trabecular number; Conn.Dn, connectivity density; Ct.Th, cortical bone thickness; Ct. Ar/Tt. Ar, cortical area fraction; ns, non-significant. AM-LD, AM low dosage; AM-HD, AM high dosage.

To explored whether pharmacological inhibition of *Ikbke* improved bone loss, we investigated the effects of amlexanox (AM), a proven IKKε inhibitor, on bone metabolism in OVX mice. Mice received daily AM or vehicle through intragastric administration for eight weeks (Fig. 2A). As expected, OVX mice gained more weight and fat compared to sham mice (Fig. 2J, 2L and Fig. S3C-F). Notably, AM treatment, particularly at high dose, reversed weight and fat gain, consistent with its known weight-loss effect. Uterine weight confirmed the successful osteoporosis model (Fig. S3A and S3B).

Three-dimensional (3D) μCT analysis revealed that AM significantly reduced bone loss in the distal femur compared to OVX group (Fig. 2D). OVX mice exhibited lower bone mass and poorer microarchitecture, as evidenced by reduced parameters including vBMD, Tb.BV/TV, Tb.BS/TV and Tb.N (Fig. 2B, 2C, 2E and 2F). High-dose AM treatment significantly improved these parameters, while the low-dose group showed trends towards improvement (Fig. 2B, 2C, 2E and 2F). AM also increased Conn.Dn in both dose groups (Fig. 2G). However, Tb.Sp, Tb.Th and SMI in AM-treated mice remained unchanged (Fig. S3G-I). For cortical bone parameters, lower Ct.Th and Ct.Ar/Tt.Ar induced by OVX were not normalized by AM (Fig. 2Η and I). Notably, AM did not show any apparent side effects in vital organs (Fig. 2K and Fig. S4).

**Figure 3:**
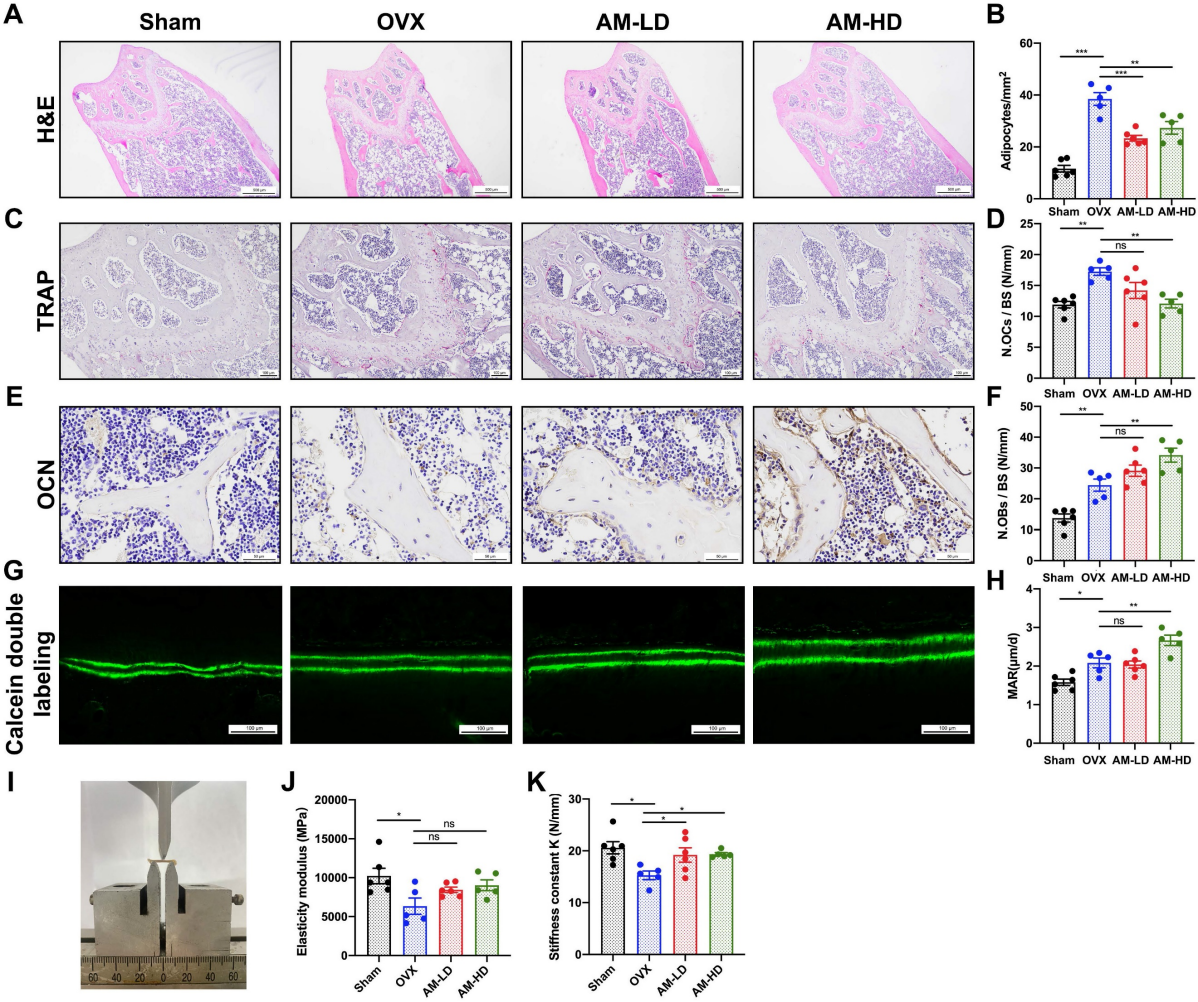
** Amlexanox enhanced bone formation, reduced marrow adiposity, and improved bone strength.** (A and B) H&E staining and quantification of adipocytes number in femora. Scale bar=500 μm. (C and D) TRAP staining and quantitative analysis of TRAP-positive cells number. Scale bar=100 μm. (E and F) Immunohistochemical staining of OCN and quantitative analysis of OCN-positive cell number. (G and H) Calcein double labeling of trabecular bone and quantitative analysis of MAR. Scale bar=100 μm. (I) Image of three-point bending test. (J and K) Mechanical properties of femora including elasticity modulus and stiffness constant K. n=5 or 6 per group. Data are presented as mean ± SD. *P < 0.05; **P< 0.01; ***P < 0.001, one-way ANOVA. OCN, osteocalcin; MAR, mineral apposition rate; TRAP, tartrate-resistant acid phosphatase.

Histological analysis revealed a significant decrease in adipocyte number with AM treatment (Fig. 3A and B). While AM-LD group displayed similar osteoblast numbers and mineral apposition rate (MAR) to OVX controls, AM-HD group exhibited a significant increase in both parameters (Fig. 3E-H). AM treatment increased bone mass and decreased bone marrow fat, correlating with enhanced bone mechanical strength (Fig. 3I). Notably, stiffness was increased in both AM groups (Fig. 3K). Other mechanical properties including elasticity modulus, maximum bending load and maximum bending strength remained unchanged (Fig. 3J and Fig. S3J-K). Furthermore, quantitative analysis of TRAP staining revealed a significantly lower number of osteoclasts in the AM-HD group compared to OVX group (Fig. 3C and 3D).

Collectively, these findings suggest that AM may act as a prophylactic agent to protect against osteoporosis by enhancing bone formation, suppressing bone resorption, and decreasing fat accumulation.

### Amlexanox led to enhanced osteogenic, inhibited adipogenic differentiation and suppressed osteoclast differentiation

**Figure 4:**
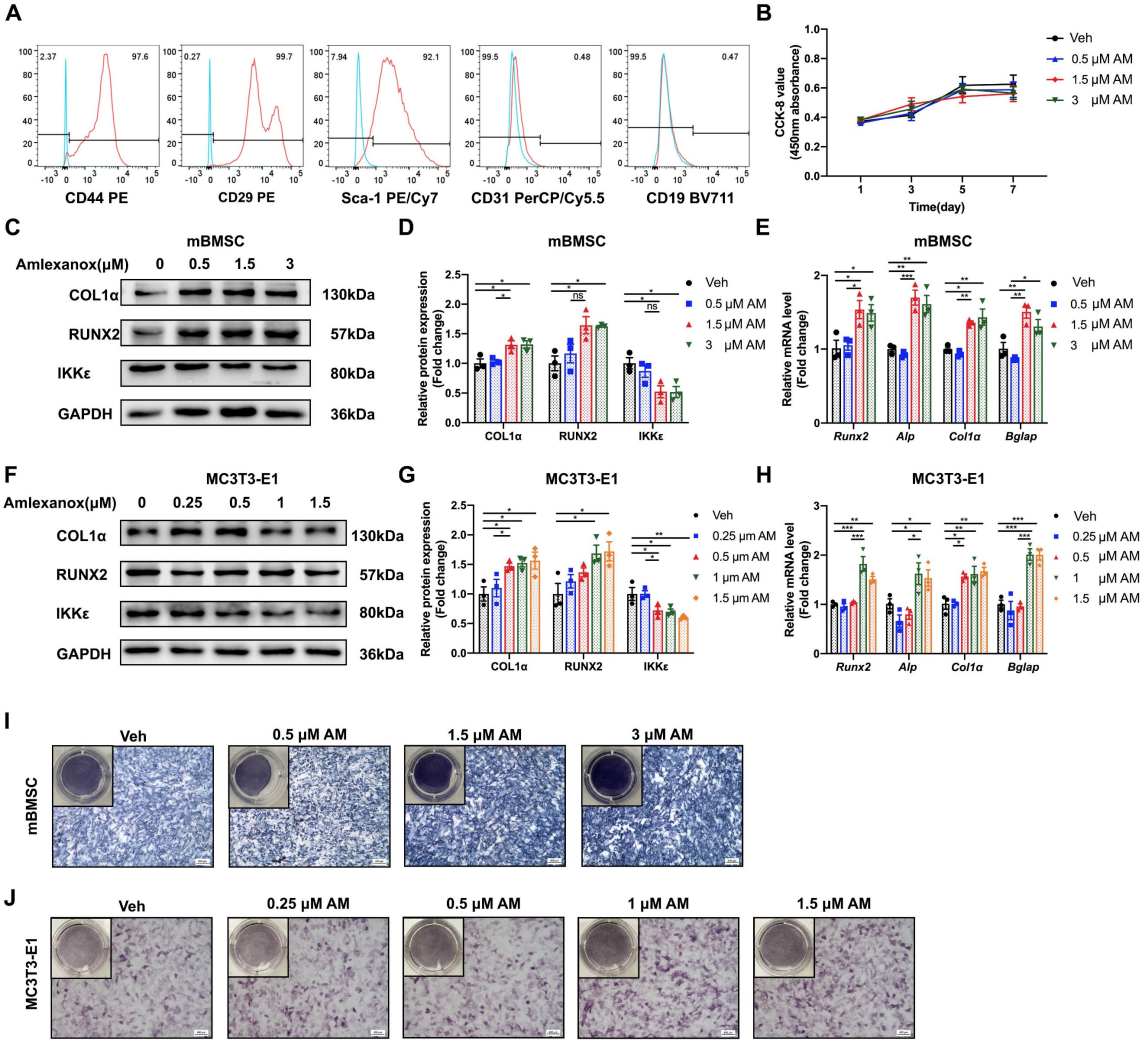
**Amlexanox promoted osteogenic differentiation of mBMSC and MC3T3-E1 in a concentration-dependent manner.** (A) Flow cytometry analysis of surface markers on mBMSCs. The test samples were represented as red curves and the controls were illustrated as blue curves. (B) Cell proliferation assay measured by CCK-8 kit. n=4 independent experiments. (C, D, F and G) Protein expression of RUNX2, COL1α, and IKKε in mBMSCs and MC3T3-E1 cells (C and F). Statistical analyses of band intensity (D and G). n=3 independent experiments. (E and H) Osteogenic markers including *Runx2*, *Alp*, *Col1α* and *Bglap* in mBMSC (E) and MC3T3-E1 (H). n=3 independent experiments. (I and J) Representative images of ALP staining in mBMSC (I) and MC3T3-E1 (J). Scale bar=400 μm. n=3 independent experiments. Data are presented as mean ± SD. *P < 0.05; **P< 0.01; ***P < 0.001, one-way ANOVA. ALP, Alkaline phosphatase.

To determine the cellular mechanisms for the increased bone mass observed in AM-treated mice, we explored the effect of AM on osteogenesis. First, we confirmed the identity of mBMSCs using flow cytometry to measure specific surface markers (CD44^+^, CD29^+^, Sca-1^+^, CD31^-^, and CD19^-^) (Fig. 4A). Also, cytotoxic effects of AM were checked by CCK8 assay. There were no significant differences in mBMSC viability at less concentration than 3 μM for 5 or 7 days (Fig. S6F). Similarly, there was no alteration of cell proliferation by AM at above concentrations (Fig. 4Β).

**Figure 5:**
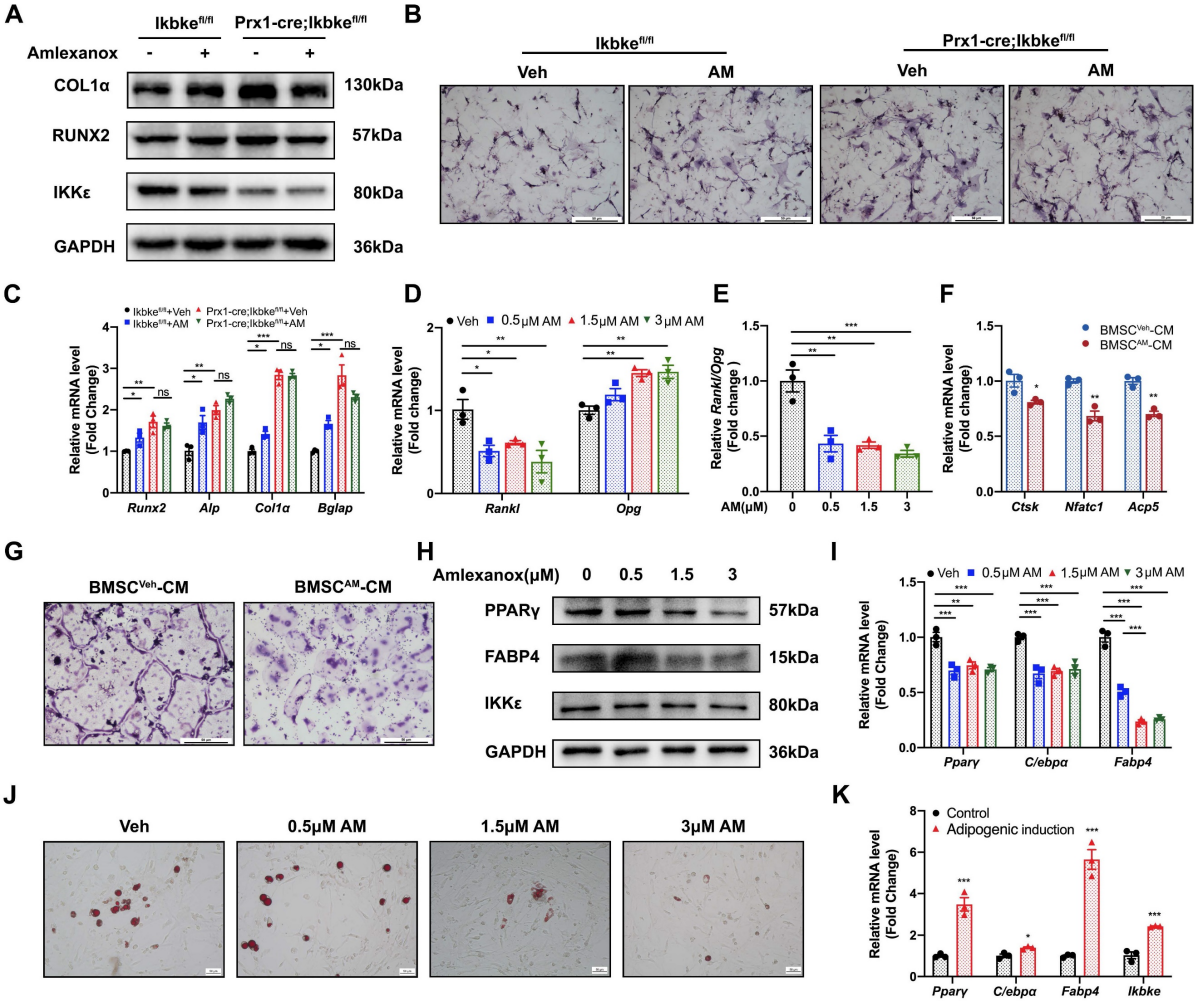
** Amlexanox through IKKε inhibition increased osteogenesis, decreased adipogenesis and suppressed osteoclast differentiation.** (A) mBMSC isolated from Ikbke^fl/fl^ and Prx1-cre; Ikbke^fl/fl^ were treated with AM followed by osteogenic induction. Protein expression of RUNX2, COL1α and IKKε were analyzed. n=3 independent experiments. (B) ALP staining after osteogenic induction for 7 days. Scale bar=50 μm. (C) Osteogenic factors measured by qRT-PCR. n=3 independent experiments. (D and E) The mRNA expression of *Rankl* and* Opg* was examined in mBMSC with series concentrations of AM treatment and the ratio of *Rankl/Opg* was calculated. n=3 independent experiments. (F) Osteoclast-specific genes of Raw 264.7 cells exposed to CM from Veh- or AM-treated BMSC was assessed by qRT-PCR. n=3 independent experiments. (G) TRAP staining. Scale bar=50 μm. (H and I) Protein and mRNA level of adipogenic genes with different doses of AM treatment. n=3 independent experiments. (J) Representative images of Oil Red O staining of mBMSCs with serial AM concentrations. Scale bar=50 μm. (K) Adipogenic genes and *Ikbke* expression were traced by qRT-PCR. n=3 independent experiments. Data are presented as mean ± SD. *P < 0.05; **P< 0.01; ***P < 0.001, one-way or two-way ANOVA. Comparisons were conducted using Student's t test for two groups. ALP, alkaline phosphatase; TRAP, tartrate-resistant acid phosphatase; CM, conditioned medium.

We next investigated the effect of AM on the osteogenic potential of mBMSCs. Increasing AM concentrations significantly enhanced the expression of osteogenic marker genes (*Runx2*, *Alp*, *Col1α*, and *Bglap*) as measured by qRT-PCR (Fig. 4E). Western blot analysis confirmed increased protein levels of RUNX2 and COL1α (Fig. 4C and 4D). Similar results were observed in MC3T3-E1, where AM treatment concentration-dependently upregulated osteogenic marker expression (Fig. 4F-H). Furthermore, there was stronger ALP staining of mBMSCs in 3 μM AM or MC3T3-E1 in 1.5 μM AM compared with vehicle (Fig. 4I and 4J). Additionally, the stimulation of osteogenic differentiation by AM was attenuated in *Ikbke* knockout mBMSCs, as evidenced by reduced protein and mRNA levels of osteogenic factors and weaker ALP staining (Fig. 5A-C and S5A). These findings suggested that AM-mediated osteogenesis promotion was dependent on IKKε expression and its regulation.

We further investigated AM's effect on adipogenesis in mBMSCs. Intriguing, IKKε expression was increased during adipogenesis (Fig. 5K), and AM treatment significantly suppressed the expression of key adipogenic genes including *Pparγ*, C/ebpα and *Fabp4* in a dose-dependent manner (Fig. 5I). This was further confirmed by reduced protein level of adipogenic markers and a marked decrease in lipid droplet formation observed with Oil Red O staining (Fig. 5H, S5B and 5J).

Overall, these findings demonstrated that AM promoted osteogenesis while simultaneously suppressing adipogenesis. This dual effect may address therapeutic potentiality for osteoporosis of AM.

### Amlexanox downregulated RANKL/OPG ratio in BMSC and inhibited osteoclast differentiation

Osteoblast Lineage can regulate the development of osteoclast through secreted factors. A critical balance exists between RANKL, which stimulates osteoclast formation, and OPG, which inhibits it^22^. Our study demonstrated that AM treatment downregulated *Rankl* mRNA expression, while upregulating *Opg* mRNA expression in mBMSC (Fig. 5D). This resulted in a significantly lower *Rankl/Opg* ratio, favoring osteoclast inhibition (Fig. 5E). Furthermore, conditioned medium (CM) collected from AM-treated mBMSCs (BMSC^AM^-CM) significantly suppressed osteoclast formation in Raw 264.7 cells, as measured by TRAP staining, compared to CM from vehicle-treated mBMSCs (BMSC^Veh^-CM) (Fig. 5G). Additionally, the expression of osteoclast-specific genes (*Nfatc1*, *Ctsk*, and *Acp5*) was decreased in Raw 264.7 cells exposed to BMSC^AM^-CM (Fig. 5F). These suggested AM controlled *Rankl* and *Opg* expression of BMSC which suppressed osteoclastogenesis indirectly.

### Amlexanox upregulated osteogenesis by reinforcing Wnt/β-catenin pathway

**Figure 6:**
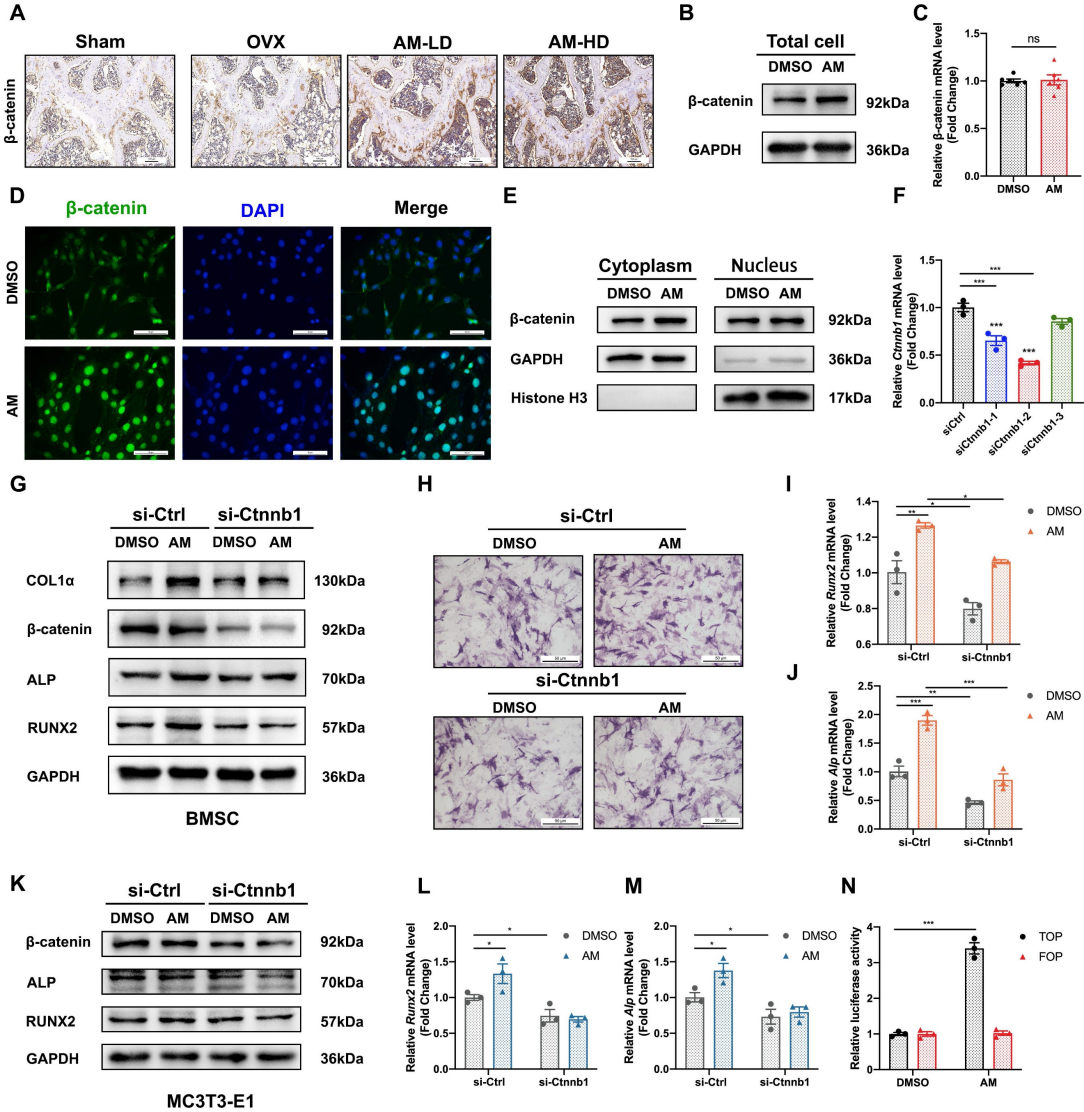
** Amlexanox upregulated osteogenesis by reinforcing Wnt/β-catenin pathway.** (A) Representative immunohistochemical staining of β-catenin in femora. Scale bar=100 μm. n=5 or 6 per group. (B) Western blot analysis revealed the levels of β-catenin protein in total cell lysates of mBMSC (n=3). (C) The mRNA level of the *Ctnnb1* gene (encoding β-catenin protein) in mBMSCs (n=3). (D) Representative immunofluorescence images showed the expression and nuclear translocation of β-catenin after AM treatment (n=3). Scale bar=50 μm. (E) β-catenin protein level in cytoplasm and nucleus after AM treatment (n=3). (F) Downregulated effect of si-Ctnnb1 confirmed by qRT-PCR (n=3). (G-J) mBMSC cells were transfected with or without si-Ctnnb1 and then treated with DMSO or AM followed by osteogenic induction for 7 days. Expression of osteogenic-related proteins was detected by western blot (G) (n=3). qRT-PCR analysis of *Runx2* (I) and *Alp* (J) (n=3). ALP staining (H) in the indicated group (n=3). (K-M) The expression of RUNX2 and ALP were assessed in control group with or without AM treatment, as well as in the si-Ctnnb1 group with or without AM treatment, following osteogenic induction for 7 days in MC3T3-E1 (K) (n=3). qRT-PCR analysis of *Runx2* (L) and *Alp* (M) in MC3T3-E1 (n=3). (N) Luciferase reporter assay showed AM treatment significantly increased the TOP flash activity (n=3). Data are presented as mean ± SD. *P < 0.05; **P< 0.01; ***P < 0.001, unpaired Student's t-test for two group's comparison and one-way or two-way ANOVA for multiple groups.

Given that the canonical Wnt/β-catenin signaling pathway plays a crucial role in regulating the balance between osteogenesis and adipogenesis, we explored whether AM regulated differentiation fate via canonical Wnt/β-catenin signaling. Histomorphometry analysis revealed increased β-catenin protein expression in bone tissues from both low-dose and high-dose AM-treated mice (Fig. 6A). Interestingly, AM treatment increased β-catenin protein levels in mBMSCs, but not the mRNA levels of the *Ctnnb1* gene (encoding β-catenin protein) (Fig. 6B, 6C and S6A). Notably, lentivirus-mediated knockdown and conditional knockout of *Ikbke* in mBMSC exhibited increased β-catenin protein levels (Fig. S6I and J). AM enhanced β-catenin protein levels in mBMSCs derived from Ikbke^fl/fl^ mice, and this increase was abolished by *Ikbke* knockout (Fig. S6J). β-catenin shuttles between the cytoplasm and nucleus, and its nuclear accumulation is a hallmark of activation. We observed that AM treatment increased β-catenin protein levels in both the cytoplasmic and nuclear fractions of mBMSCs (Fig. 6E and S6D). Immunofluorescence analysis further confirmed nuclear accumulation of β-catenin with AM treatment (Fig. 6D). To assess the transcriptional activity of increased nuclear β-catenin, we used paired TOP/FOP-Flash luciferase reporter assays. These assays demonstrated a significant increase in β-catenin activity in response to AM treatment in C3H/10T1/2 MSCs and 293T cells (Fig. 6N and Fig. S6E).

To definitively link Wnt/β-catenin signaling with AM, we used siRNA to knock down β-catenin expression in mBMSCs and MC3T3-E1 cells. β-catenin knockdown significantly decreased *Ctnnb1* mRNA levels (Fig. 6F). Subsequently, β-catenin-knockdown mBMSCs were treated with AM during osteogenic induction. This treatment blocked AM-induced increases in RUNX2, ALP, and COL1α protein expression (Fig. 6G and S6B) and mRNA levels of *Runx2* and *Alp* (Fig. 6I and 6J). Additionally, stronger ALP staining observed with AM treatment was suppressed by β-catenin knockdown (Fig. 6H). Similar results were obtained in MC3T3-E1 cells, where β-catenin silencing abrogated AM's stimulatory effect on osteogenic differentiation at both the protein and mRNA levels (Fig. 6K-M and S6C).

Taken together, these data indicated that AM promoted osteogenesis through a post-transcriptional activation of the Wnt/β-catenin signaling pathway.

### Amlexanox suppressed ubiquitin-proteasome dependent degradation of β-catenin

**Figure 7:**
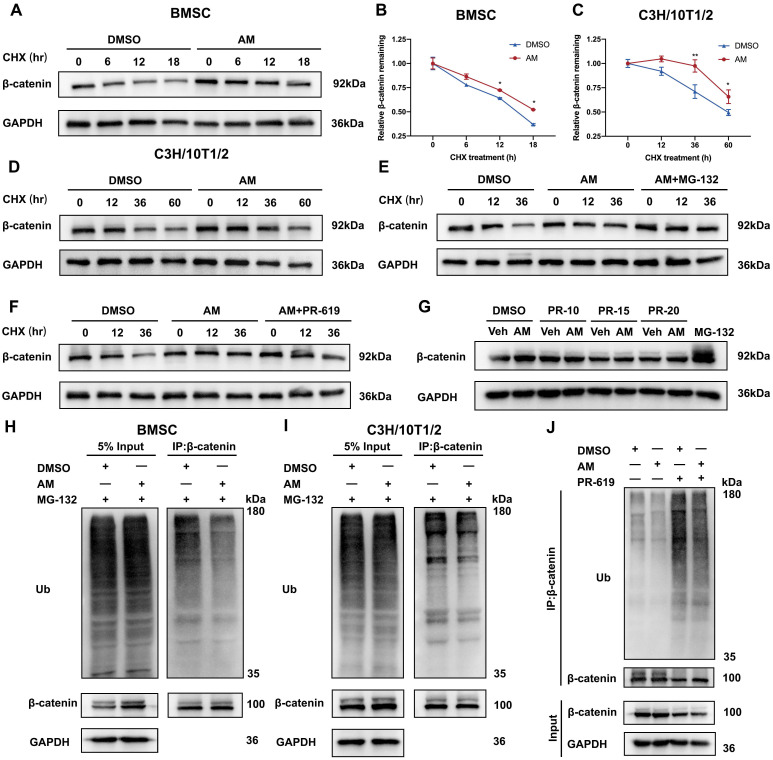
** Amlexanox suppressed ubiquitin-dependent proteasomal degradation of β-catenin**. (A-D) Western blot analysis and quantification were conducted to evaluate the impact of AM on β-catenin stability in mBMSC and C3H/10T1/2 cells incubated with cycloheximide (CHX) at different time points (n=3). (E and F) C3H/10T1/2 cells pre-treated with AM were intervened with CHX in the presence of MG132 (20 mM) or PR-619 (15 μM). n=3 independent experiments. (G) C3H/10T1/2 cells were treated with or without AM and co-incubated with different dosage gradients of PR-619 for 48 hours (n=3). (H and I) mBMSC and C3H/10T1/2 cells were treated with or without AM treatment. After 8h of MG132 treatment, the cell lysates were treated with β-catenin antibody for IP and ubiquitination was detected (n=3). (J) C3H/10T1/2 cells were co-incubated with or without AM in the presence of PR-619 for 48 hours, followed by IP with anti-β-catenin and IB with indicated antibodies (n=3). Data are presented as mean ± SD. *P < 0.05; **P< 0.01; ***P < 0.001, unpaired Student's t-test.

To investigate how AM regulates β-catenin protein levels, we examined its stability in mBMSCs and C3H/10T1/2 cells. Blocking new protein synthesis with cycloheximide (CHX) revealed that AM treatment significantly extended β-catenin protein half-life in both cell types (Fig. 7A-D). We further explored the mechanism by using MG132, a proteasome inhibitor. MG132 treatment increased β-catenin protein levels in AM-treated cells compared to controls (Fig. 7E). Conversely, treatment with 3-Methyladenine (3-MA), a lysosome inhibitor, did not affect β-catenin levels (Fig. S6H). These findings suggested that AM promoted β-catenin stability through the ubiquitin-proteasome pathway. Supporting this notion, we observed that AM treatment significantly decreased the ubiquitinated β-catenin protein in both mBMSCs and C3H/10T1/2 cells (Fig. 7H and 7I). Notably, this decrease was abolished by PR-619, an inhibitor of deubiquitinating enzymes (Fig. 7J). Furthermore, treatment with PR-619 not only reversed the AM-mediated extension of β-catenin half-life (Fig. 7F) but also increased its ubiquitination and attenuated the AM-induced upregulation of β-catenin protein in a dose-dependent manner (Fig. 7G and Fig. S6G). In conclusion, these findings demonstrated that AM stabilized β-catenin protein by suppressing its ubiquitin-dependent degradation in MSCs.

### Amlexanox promoted osteogenesis in human BMSCs (hBMSCs)

**Figure 8:**
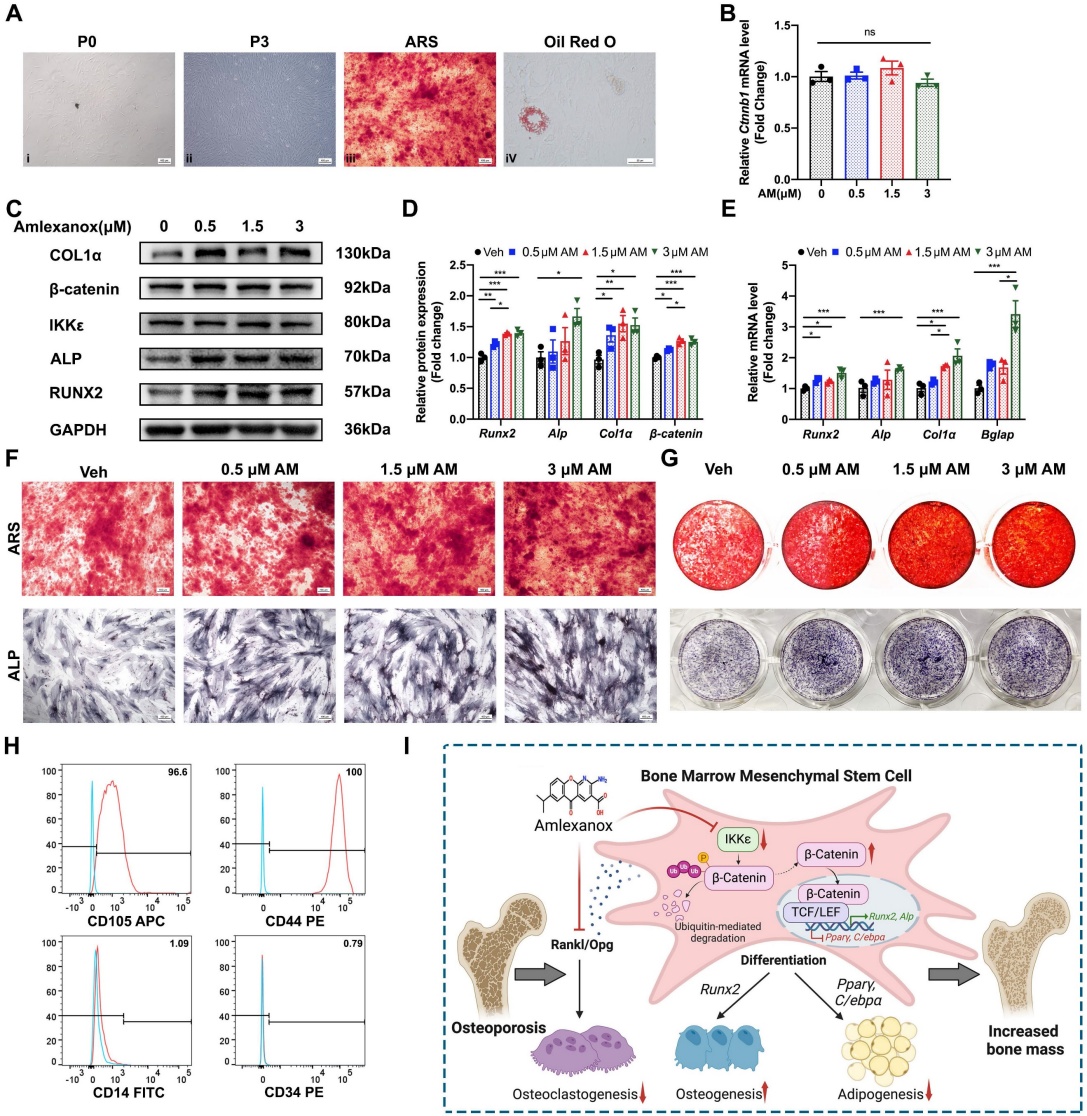
** Amlexanox regulated osteogenesis in human BMSCs (hBMSCs).** (A) hBMSC exhibited uniform and long spindle-shaped cells in passages 0 (**i**; Scale bar=400 μm) and passages 3 (**ii**; Scale bar=400 μm). hBMSCs could differentiate into osteoblasts and adipocytes, as detected by ARS (**iii**; Scale bar=400 μm) and Oil Red O staining (**iv**; Scale bar=50 μm). (B)The mRNA level of *Ctnnb1* (n=3). (C and D) Protein expression and quantification of osteogenic marker genes, IKKε and β-catenin (n=3). (E) The mRNA level of osteogenic markers (n=3). (F and G) Representative images of ALP and ARS staining. Scale bar=400 μm. (H) Flow cytometry analysis of surface markers on mBMSCs (passages 3). The test samples were represented as red curves and the controls were illustrated as blue curves. (I) The schematic diagram showed that AM promoted osteogenesis while suppressing adipogenesis and osteoclastogenesis. This effect was mediated by inhibiting β-catenin degradation via the ubiquitin-proteasome system in MSCs, ultimately leading to improved bone mass and microarchitecture. Data are presented as mean ±SD. *P < 0.05; **P< 0.01; ***P < 0.001, one-way ANOVA. ARS, alizarin red S; ALP, alkaline phosphatase.

To assess AM's potential for improving bone mass in humans, we investigated its effects on hBMSCs. hBMSCs displayed a uniform, elongated spindle-shaped morphology under an optical microscope (Fig. 8A). After treatment with osteogenic or adipogenic inducers, hBMSCs formed mineralized nodules (by ARS stain) and lipid droplets (by Oil Red O stain), confirming their differentiation potential (Fig. 8A). Analysis of surface markers revealed that over 95% of hBMSCs were positive for CD44 and CD105, markers characteristic of MSC, while less than 2% expressed CD34 and CD14 (Fig. 8H). Similar to the effects observed in mouse BMSCs (mBMSCs), AM treatment in hBMSCs significantly increased the expression of osteogenic marker genes (Fig. 8E). Western blot analysis further confirmed increased protein levels of RUNX2, ALP, and COL1α (Fig. 8C and 8D). Interestingly, AM treatment elevated β-catenin protein levels without affecting its mRNA levels (Fig. 8B-D), consistent with our observations in mBMSCs. Furthermore, ALP staining and mineralized nodule formation, indicators of osteogenic potential, exhibited a dose-dependent increase with AM treatment, reaching a significant difference at 3 μM (Fig. 8F and 8G).

In summary, AM promoted osteogenesis and suppressed adipogenesis by inhibiting the ubiquitin-dependent degradation of β-catenin in MSCs, thereby increasing bone mass and bone microarchitecture (Fig. 8I).

## Discussion

Osteoporosis is a debilitating disease characterized by low bone mass, weakened bone microarchitecture, and increased risk of fractures^23^. Current treatments for osteoporosis include anabolic agents and antiresorptive agents^24^. Anabolic agents like teriparatide and abaloparatide increase osteoblast numbers and bone formation, but they also stimulate osteoclasts to drive coupled remodeling^25^. Long-term medication of antiresorptive agents such as bisphosphonates and denosumab can weaken bone strength by inhibiting the release of factors that couple bone formation with resorption^26^. Romosozumab, a newly-approved drug with the desirable dual effect of promoting bone formation and inhibiting resorption, carries a potential risk of adverse cardiovascular events^27^. Therefore, there is an urgency for developing novel drugs that can effectively both stimulate bone formation and inhibit bone resorption. In the current study, we investigated the effects of AM on osteogenesis and bone homeostasis both* in vitro* and *in vivo*.

The data revealed IKKε was decreased during osteoblast differentiation. Moreover, IKKε was upregulated in the OVX-derived mBMSCs and hBMSCs from osteoporosis donors, suggesting its physiological role in osteoblast lineage cells. Moreover, IKKβ which is highly homologous to IΚΚε regulated MSC differentiation^28^, indicating the involvement of IΚΚε in MSC osteoblast differentiation. In loss-of-function study, we confirmed the knockdown of *Ikbke* in mBMSCs stimulated osteogenesis. Based on these findings, we investigated whether pharmacological inhibiting of IKKε could promote bone formation.

We explored AM, a known IKKε inhibitor, for its effects on bone metabolism. AM treatment significantly reduced bone loss and improved bone microarchitecture in OVX mice, particularly at the higher dose (100 mg/kg). This dosage difference compared to a previous study (20 mg/kg) might be due to variations in mouse age and administration methods^29^. AM increased bone mass and formation while reducing bone marrow fat. Notably, the newly formed bone exhibited high biomechanical strength in bending tests. Our study showed that AM increased the number and function of osteoblasts, as evidenced by increased OCN-positive cells and MAR. Furthermore, AM reduced the number of osteoclasts, as indicated by TRAP staining, consistent with previous research^29^.

Our *in vitro* experiments confirmed that AM had multiple beneficial effects in BMSCs: (1) Enhanced osteogenic differentiation and inhibited adipogenic differentiation: AM promoted the development of BMSCs into osteoblasts and suppressed their differentiation into adipocytes, favoring bone formation over fat accumulation in a dose-dependent manner. This effect was diminished in BMSCs lacking *Ikbke*, suggesting a IΚΚε dependance of AM action. (2) Suppressed osteoclastic differentiation: AM also regulated the production of RANKL and OPG from BMSCs, two crucial factors involved in osteoclast development. This suggests that AM might inhibit osteoclast formation through both direct and indirect mechanisms, potentially including the NF-κB and mitogen-activated protein kinase (MAPKs) pathways as reported previously^29^.

Previous studies have demonstrated the importance of Wnt/β-catenin signaling in regulating the osteogenesis and adipogenesis of MSCs^30^. Αctivation of Wnt/β-catenin signaling suppressed expression of CCAAT/enhancer-binding protein alpha (C/ebpα) and peroxisome proliferator-activated receptor gamma (Pparγ), while increasing the expression of Runx2, distal-less homeobox 5 (Dlx5), and Οsterix^31^, ^32^. Previous studies have even shown that the loss of β-catenin in preosteoblasts can lead these cells to differentiate into adipocytes instead of osteoblasts^33^. Our findings supported this connection between Wnt/β-catenin signaling and the effect of AM. AM increased the total and nuclear protein levels of β-catenin, suggesting the activation of β-catenin signaling. Furthermore, AM increased transcriptionally active nuclear β-catenin, as measured by TOP/FOP-Flash luciferase reporters. Importantly, silencing β-catenin blocked the stimulatory effect of AM on osteogenic factors. This data strongly suggested that the effect of AM to influence cell fate decisions in BMSCs relied on its activation of the Wnt/β-catenin pathway. Interestingly, a recent study identified AM as a novel Wnt/β-catenin activator which could induce lung epithelial organoid formation, making it a potential therapeutic agent for emphysema^34^.

Our study revealed that AM increased β-catenin protein levels without affecting its mRNA levels, suggesting a post-transcriptional mechanism. It was found that AM could increase the stability of β-catenin and prolong its protein half-life using CHX chase method and MG132 assay. Furthermore, *in vivo* ubiquitination assays showed that AM attenuated the ubiquitination level of β-catenin, which was abolished by a deubiquitinase inhibitor. Collectively, these findings indicated that AM regulated β-catenin expression by inhibiting its ubiquitination-proteasome degradation. Previous research has shown that IKKε phosphorylates β-catenin, thereby inhibiting its downstream signaling activation^35^. Additionally, IKKβ phosphorylated the serine-33, -37, and -45 of β-catenin, regulating its ubiquitination in MSCs^28^. Based on these findings and the influence of AM on protein posttranslational regulation and ubiquitination, we hypothesized that IKKε might regulate β-catenin degradation through ubiquitination. However, there is a great need of further studies to elucidate the precise role of IKKε in this process.

In previous studies, AM was proved to inhibit cell proliferation in prostate tumors and glioblastoma^10^, ^36^, involved in lung epithelial cells repairing in emphysema^34^, lipid oxidation for obesity^16^, adipose-liver signaling axis in type 2 diabetes^15^, and attenuating neuralgia^37^. These diverse pharmacological effects were all linked to the inhibition or deficiency of IKKε or TBK1. Given the high homology (82.9%) between human and mouse IKKε proteins, we investigated the effect of AM on hBMSCs. AM promoted osteogenic differentiation of hBMSC in a dose-dependent manner while upregulating β-catenin protein, mirroring the mechanism observing in mBMSCs. These results suggested AM as a promising candidate drug for osteoporosis treatment in near future.

In brief, this study demonstrated a novel effect of AM: rebalancing bone homeostasis by promoting osteogenesis while inhibiting adipogenesis and osteoclastogenesis (Fig. 8I). This resulted in an overwhelming advantage of bone formation over resorption. Mechanistically, AM achieved this effect by specifically inhibiting IKKε, thereby suppressing the ubiquitination and subsequent degradation of β-catenin in MSCs. Given the established safety profile of AM, these findings suggest its potential as a therapeutic candidate for osteoporosis.

## Supplementary Material

Supplementary figures and table.

## Figures and Tables

**Table 1:** Sequences of designed primers for murine genes

Gene	Sense	Sequence (5'-3')	Gene	Sense	Sequence (5'-3')
*Ikbke*	FWD	CCCAGGAGATGCAGAGTACC	*Pparγ*	FWD	CATCAGGCTTCCACTATG
	REV	CCACCTCCCCGGATTTCTTG		REV	CACAGCAAGGCACTTCTG
*Ikbka*	FWD	GTCAGGACCGTGTTCTCAAGG	*C/ebpα*	FWD	ACTCCTCCTTTTCCTACCG
	REV	GCTTCTTTGATGTTACTGAGGGC		REV	AGGAAGCAGGAATCCTCC
*Ikbkb*	FWD	GAGCTCAGCCCAAAGAACAG	*Fabp4*	FWD	AAGGTGAAGAGCATCATAACCCT
	REV	AGGTTCTGCATCCCCTCTGG		REV	TCACGCCTTTCATAACACATTCC
*Runx2*	FWD	GACGTGCCCAGGCGTATTTC	*Ctnnb1*	FWD	GTACGCACCATGCAGAATAC
	REV	AAGGTGGCTGGGTAGTGCATTC		REV	TGGAGCAGGAGATTATGCAG
*Col1α*	FWD	TGACTGGAAGAGCGGAGAGT	*Ctsk*	FWD	GCTTGGCATCTTTCCAGTTTTA
	REV	GTTCGGGCTGATGTACCAG		REV	CAACACTGCATGGTTCACATTA
*Alp*	FWD	CCAACTCTTTTGTGCCAGAGA	*Nfatc1*	FWD	GAGGAACACGCTGATGCC
	REV	GGCTACATTGGTGTTGAGCTTTT		REV	AGGCGAGTTGGGTTGGAT
*Bglap*	FWD	GCCGGAGTCTGTTCACTACC	*Acp5*	FWD	TACCTGTGTGGACATGACC
	REV	GCGCTCTGTCTCTCTGACCT		REV	CAGATCCATAGTGAAACCGC
*Rankl*	FWD	CCAAGATCTCTAACATGACG	*Opg*	FWD	CAGAGCGAAACACAGTTTG
	REV	CACCATCAGCTGAAGATAGT		REV	CACACAGGGTGACATCTATTC
*Gapdh*	FWD	AACTTTGGCATTGTGGAAGG			
	REV	ACACATTGGGGGTAGGAACA			

**Table 2:** Sequences of designed primers for human genes

Oligo name	Sense	Sequence (5'-3')
*RUNX2*	FWD	AGGCAGTTCCCAAGCATTTCATCC
	REV	TGGCAGGTAGGTGTGGTAGTGAG
*COL1α*	FWD	TGGCAAAGAAGGCGGCAAAGG
	REV	AGGAGCACCAGCAGGACCATC
*BGLAP*	FWD	AGGGCAGCGAGGTAGTGAAGAG
	REV	GGTCAGCCAACTCGTCACAGTC
*ALP*	FWD	GGACATGCAGTACGAGCTGA
	REV	GCAGTGAAGGGCTTCTTGTC
*CTNNB1*	FWD	GGCTCTTGTGCGTACTGTCCTTC
	REV	CTTGGTGTCGGCTGGTCAGATG
*GAPDH*	FWD	GGAGCGAGATCCCTCCAAAAT
	REV	GGCTGTTGTCATACTTCTCATGG

## Abbreviations

AM: Amlexanox
ALP: alkaline phosphatase
ARS: alizarin red S
BMSC: bone marrow mesenchymal stem cell
CTSK: cathepsin K
C/EBPα: CCAAT/enhancer-binding protein alpha
COL1α: type 1 collagen alpha
CCK8: cell counting kit-8
CM: conditioned medium
CHX: Cycloheximide
DLX5: distal-less homeobox 5
EDTA: ethylene-diamine tetraacetic acid
FABP4: Fatty acid-binding protein 4
IKKε: inhibitor of nuclear factor κB kinase epsilon
IFN: interferon
IRF: interferon regulatory factor
LPS: lipopolysaccharide
MAPK: mitogen-activated protein kinase
MAR: mineral apposition rate
3-MA: 3-Methyladenine
Nfatc1: nuclear factor of activated T-cells
NAFLD: non-alcoholic fatty liver disease
NF-κB: nuclear factor-κB
OCN: osteocalcin
OPG: Osteoprotegerin
OVX: ovariectomy
PPARγ: peroxisome proliferator-activated receptor gamma
P/S: penicillin and streptomycin. PBS: Phosphate Buffer Saline
PVDF: polyvinylidene difluoride
RA: rheumatoid arthritis
ROI: the region of interest
Runx2: runt-related transcription factor 2
RANKL: receptor activator for nuclear factor κB ligand
STAT1: signal transducer and activator of transcription 1
TRAP: tartrate-resistant acid phosphatase
TBK1: TANK-binding kinase 1
UCP1: uncoupling protein 1
