# Supplementary material for: Amlexanox Enforces Osteogenic Differentiation and Bone Homeostasis Through Inhibiting Ubiquitin-Dependent Degradation of β-Catenin
Source: Int J Biol Sci. 2024 Sep 30;20(13):5254–71. doi: 10.7150/ijbs.101507 (PMC11489180; doi:10.7150/ijbs.101507)
Supplement: Supplementary file 1 — Supplementary figures and table. [file ijbsv20p5254s1.pdf]

Supplementary Materials

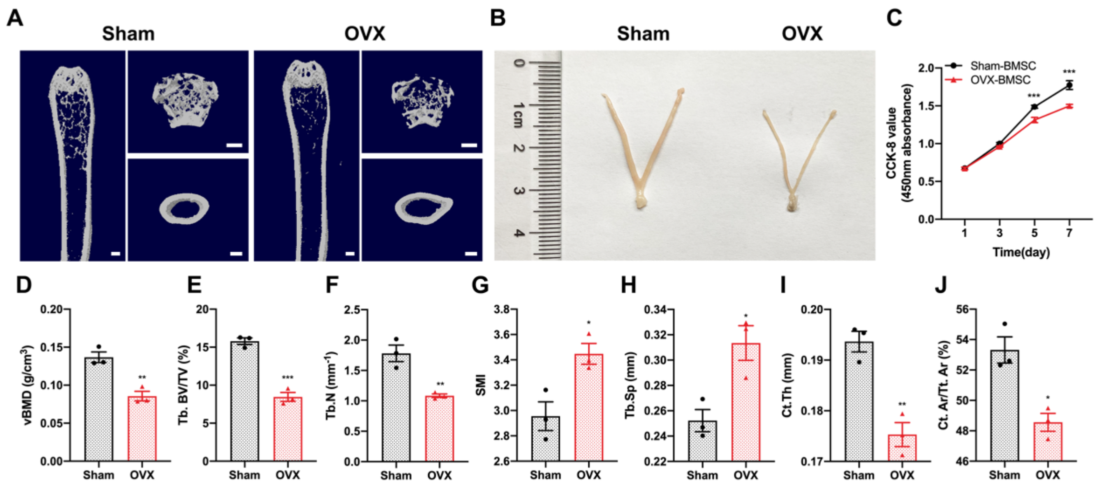

Fig. S1. OVX mice exhibited bone loss and impaired proliferation ability. (A) Representative μCT images. Scale bar= 500 μm. n=3 per group. (B) Representative images of uterus in sham and OVX group. (C) Cell proliferation assay measured by CCK-8 kit. (D-J) Quantitative analyses of parameters regarding bone microstructure and cortical bone, including vBMD, Tb.BV/TV, Tb.N, SMI, Tb.Sp, Ct. Th and Ct. Ar/Tt. Ar. Data are presented as mean ± SD. *P < 0.05; **P< 0.01; ***P < 0.001, unpaired Student's t-test. vBMD, volumetric bone mineral density; Tb. BV/TV, trabecular bone volume fraction; Tb.N, trabecular number; SMI, structure model index; Tb.Sp, trabecular spacing; Ct.Th, cortical bone thickness; Ct. Ar/Tt. Ar, cortical area fraction.

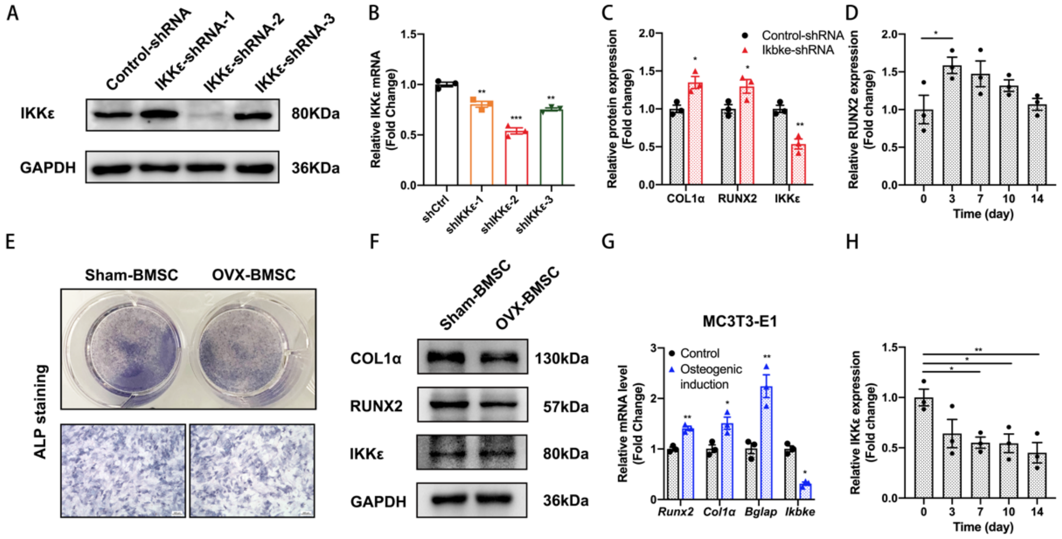

Fig. S2. IKKε expression was upregulated in OVX-derived mice.

(A and B) The knockdown of *Ikbke* was verified using western blot and qRT-PCR in mBMSCs after infection of Ikbke-shRNA expression lentivirus. (C) Statistical analyses of osteogenic protein expression including COL1α and RUNX2 in Ikbke-knockdown mBMSCs (n=3). (D and H) Statistical analyses of RUNX2 and IKKε protein expression in mBMSCs during osteogenesis differentiation (n=3). (E) ALP staining of mBMSC from sham and OVX mice. Scale bar=400 μm. (F) Protein levels of osteogenic factors including COL1α, RUNX2, and IKKε expression in mBMSC from sham or OVX mice after osteogenic induction (n=3). (G) Osteogenic genes and *Ikbke* were measured by qRT-PCR in osteoblastic cell line MC3T3-E1. Data are presented as mean ± SD. *$P < 0.05$; **$P < 0.01$; ***$P < 0.001$, one-way ANOVA or unpaired Student's t-test. OVX, ovariectomy.

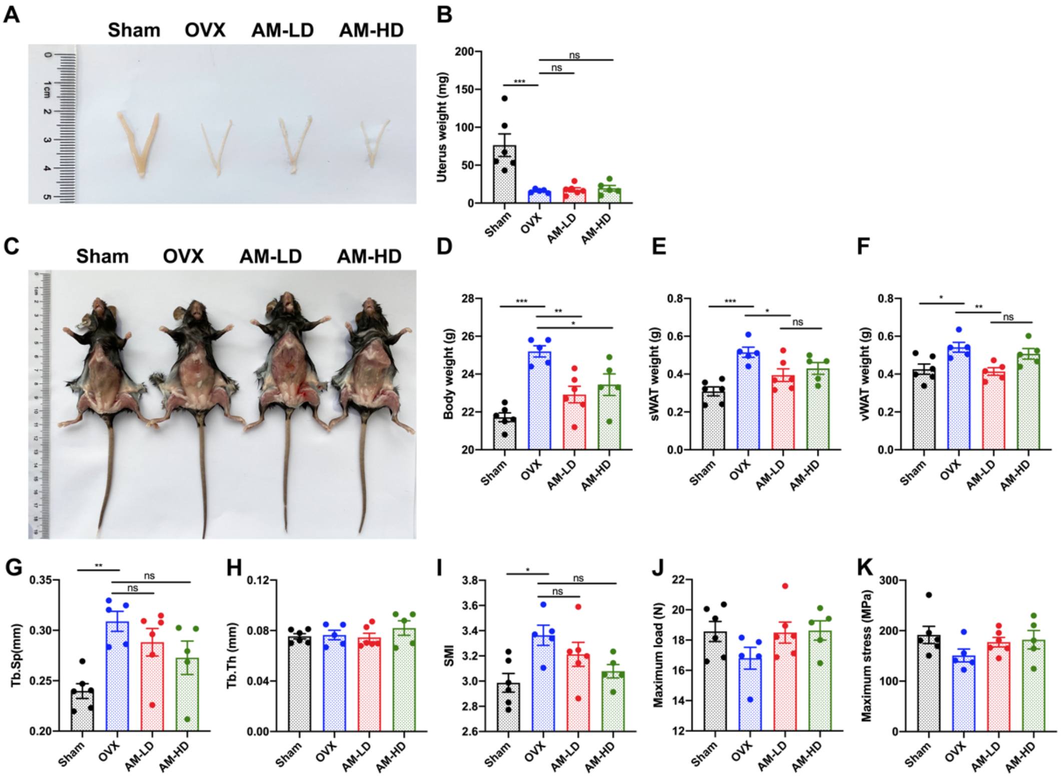

Fig. S3. Amlexanox reversed OVX-induced weight gain. (A) Uterus shown in representative images of sham, OVX and OVX mice subjected to daily intragastric administration of AM at different dosages for 8 weeks. (B) Uterus weight in each group. (C) Adipose tissue shown in representative images in each group. (D-F) Body weight (D), subcutaneous adipose tissue (sWAT) (E), and visceral adipose tissue (vWAT) (F) in indicated groups. (G-I) Quantitative analyses of parameters regarding bone microstructure including Tb.Sp (G), Tb.Th (H) and SMI (I). (J and K) Femora biomechanical properties including the maximum bending load (J) and maximum bending strength (K). Data are presented as mean ± SEM. **P* < 0.05; ***P*< 0.01; ****P* < 0.001, one-way ANOVA. n = 5 or 6 per group. Tb.Sp, trabecular spacing; Tb.Th, trabecular thickness; SMI, structure model index; ns, non-significant; AM-LD, AM low dosage; AM-HD, AM high dosage.

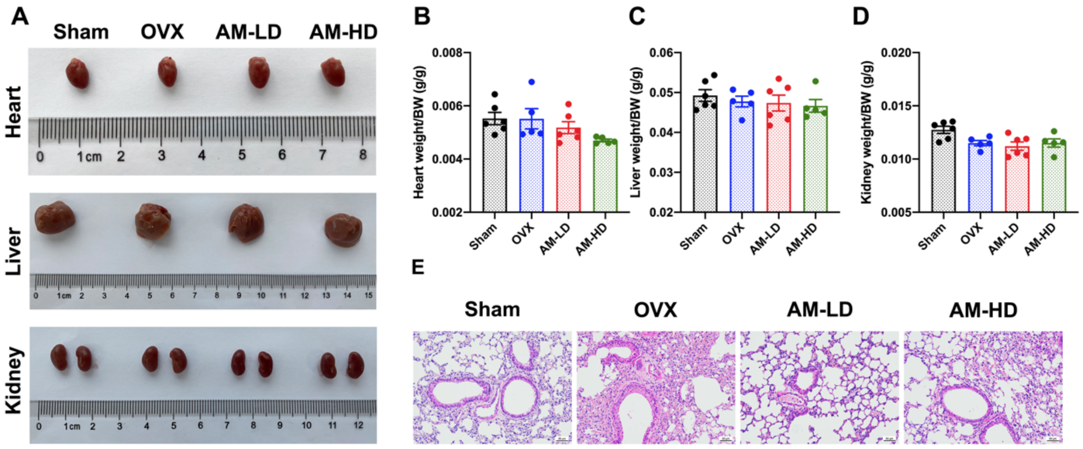

Fig. S4. Amlexanox did not cause tissue damage. (A) Representative images of heart, liver and kidney tissues in different groups with AM treatment. (B-D) Heart weight (B), liver weight (C) and kidney weight (D) per gram body weight in each group. (E) H&E staining of lung tissue after AM treatment with different dosages for 8 consecutive weeks. Scale bar = 50μm. Data are presented as mean ± SEM. n = 5 or 6 per group.

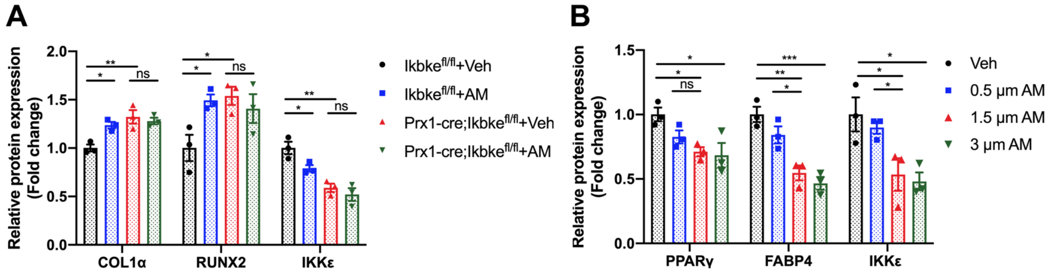

Fig. S5. Amlexanox through IKKε inhibition increased osteogenesis and decreased adipogenesis. (A) mBMSC isolated from $Ikbke^{fl/fl}$ and Prx1-cre; $Ikbke^{fl/fl}$ were treated with AM followed by osteogenic induction. Statistical analyses of protein expression including RUNX2, COL1α and IKKε were shown. n=3 independent experiments. (B) Statistical analyses of adipogenic genes protein expression with different doses of AM treatment. n=3 independent experiments. *$P < 0.05$; **$P < 0.01$; ***$P < 0.001$, one-way or two-way ANOVA.

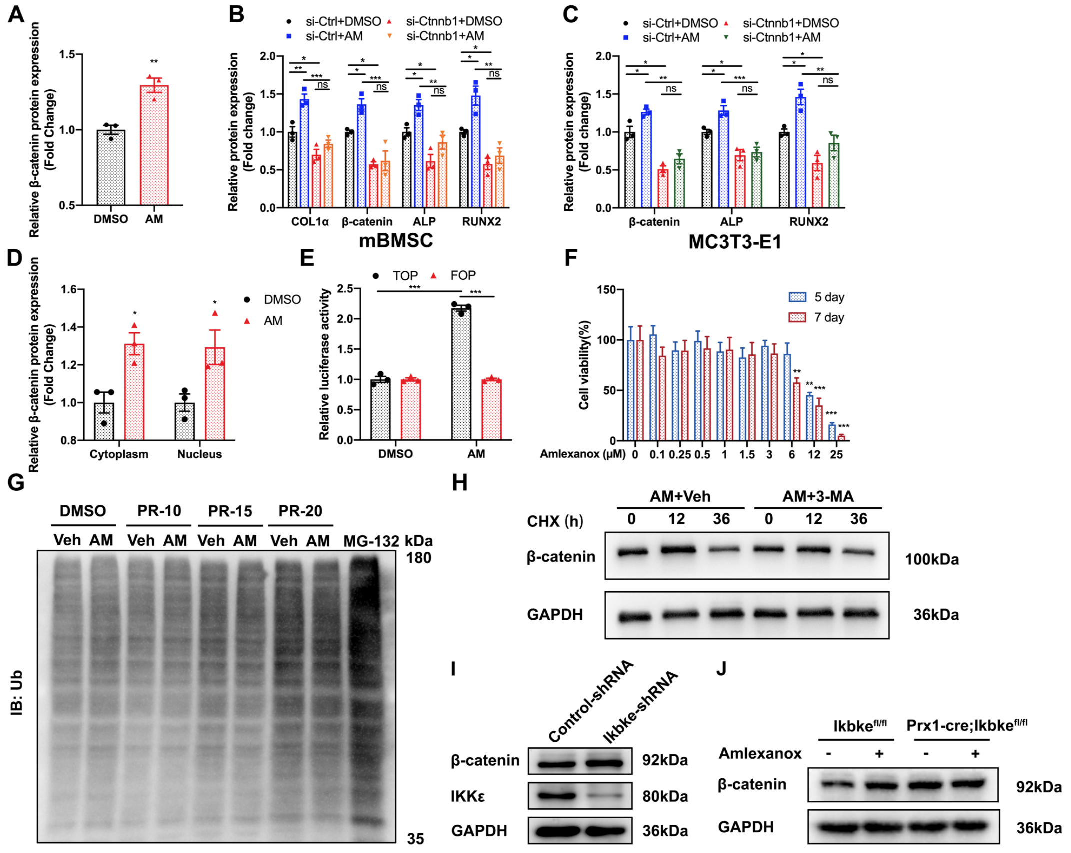

Fig. S6. Amlexanox reinforced Wnt/β-catenin pathway. (A) Statistical analyses of β-catenin protein expression in total cell lysates of mBMSC after AM treatment (n=3). (B and C) mBMSC and MC3T3-E1 cells were transfected with or without si-Ctnnb1 and then treated with DMSO or AM followed by osteogenic induction for 7 days. Statistical analyses of osteogenic-related proteins in mBMSC (B) and MC3T3-E1 cells (C) (n=3). (D) Statistical analyses of β-catenin protein level in cytoplasm and nucleus after AM treatment (n=3). (E) TOP/FOP flash luciferase reporter assay in 293T (n=3). (F) CCK-8 analysis was performed with series concentrations of AM in mBMSCs for 5 days and 7 days. n = 4 biologically independent samples. *$P < 0.05$;**$P < 0.01$; ***$P < 0.001$, compared with the pure culture medium group. (G) C3H/10T1/2 cells were treated with or without AM and co-incubated with different dosage gradients of PR-

619 for 48 hours, followed by IB of ubiquitination antibody (n=3). (H) C3H/10T1/2 cells pre-treated with AM were co-incubated with CHX in the presence or absence of 3-MA (5 mM). n=3 independent experiments. (I) Western blot analysis revealed the levels of β-catenin protein in control and *Ikbke* knockdown mBMSC (n=3). (J) mBMSC isolated from Ikbke$^{fl/fl}$ and Prx1-cre; Ikbke$^{fl/fl}$ were treated with AM followed by osteogenic induction. β-catenin protein were measured by western blot (n=3). Data are presented as mean ± SD. **P* < 0.05; ***P*< 0.01; ****P* < 0.001, unpaired Student's t-test for two group's comparison and two-way ANOVA followed by a Bonferroni's post hoc test for multiple group.

Supplementary Table 1 Baseline characteristics of individual human subjects.

Subject	Age（year）	Gender	T score (L1-L4)
1	54	Female	-2.8/-1.7/-1.8/-1.6/-1.7
2	66	Female	-3.2/-2.6/-1.7/-1.2/-1.8
3	65	Female	-3.6/-2.9/-2.5/-2.5/-2.6
4	64	Female	-2.8/-2.3/-0.5/-2.9/-1.8
5	57	Female	-3.9/-3.0/-2.5/-1.3/-2.2
